# Mr. Ring, on Dr. Rowley's Pamphlet

**Published:** 1806-02

**Authors:** John Ring

**Affiliations:** New Street, Hanover Square


### *To the Editors of the Medical and Physical Journal.*

GENTLEMEN,

IN my last communication, I made a few remarks on Dr. Rowley's ox-faced boy; in my present I propose to give a short account of his mangey girl. These two elegant and delicate portraits, to use the language of Dr. Rowley, grace the head of his tract, and serve as a double frontispiece. The latter, as well as the former, is a bare-faced imposition; on which the following motto should be inscribed, *Fronti nulla fides.*

It is a little remarkable, that in the first edition of Dr. Rowley's publication, the face is represented free from blemish; but in the second edition, there are ulcerations on the face; and it is probable that in time we shall see them spread over the whole body. Hence it may appear to the public, that the disorder is worse than when the first edition of the pamphlet was published. This, however, is far from being the case: on the contrary, there is not at present a single sore remaining. Even at the time when Dr. Rowley saw her, she had none on the face.

According to his statement, she was vaccinated in April 1803, and the complaint in question did not appear till June 1804. Had it been occasioned either by the cow-pock, as Dr. Rowley supposes, or by any other disease communicated at the same time, it is impossible it could have lurked in the habit so long. It was, in fact, neither more nor less than a case of scald head; which, not being properly treated in the beginning, spread over several parts of the body, where the matter was applied; a circumstance which is extremely common. In consequence of this, swellings took place in the inguinal glands, which still remain. This case therefore, as well as the former, is evidently scrofulous.

Dr. Rowley says, the child was a mere Lazarus, covered all over with sores and disease. He takes care to inform his his readers that he is prescribing for her his *mineral alteratives*; but he takes care not to inform them, that Mr. Beveridge, of Newgate-street, had previously been prescribing for her *his* mineral alteratives. The truth is, the child had been for some time under the care of Mr. Beveridge, and both Mr. Beveridge and the mother assure me, that all the sores, except one or two, were healed before Dr. Rowley saw her. A lodging was taken for her at Kentish Town, in order to restore her strength; and when her mother was conveying her thither in the stage, she was advised by a lady and a gentleman to shew her to Dr. Rowley. The consequence was, that the Doctor prescribed for this unfortunate, and, he truly adds, *very unfortunate*, object. In the first place, she was so unfortunate as to go through one long course of mineral alteratives; and in the next place, when reduced to a state of extreme debility, she was so unfortunate as to go through a second. Having long ago fully expressed, in this Journal, my opinion of the impropriety of giving mercurials in cases of scald head, it is the less necessary to enlarge on that subject here. I shall only observe, that many instances have verified the truth of this opinion, both before and since. The same observation may be applied to other scrophulous cases.

In the present instance, the very judicious external application employed by Mr. Beveridge, healed the ulcers. It consisted of equal parts of Ung. Hydrarg. Nitr. and Ung. Ceræ. As to the scrofulous disposition, it is the general opinion of medical practitioners, that it is not to be removed by mineral alteratives, which increase debility, but by the tonic plan; by country air, a nutritive diet, sea-bathing, bitters, and steel.

Dr. Rowley pretends, that the abscesses, in such cases as these, contain a fluid dissimilar *to* any other; and he will be able to prove, that they contain matter dissimilar *from* any others when he has proved, that a child who has undergone vaccination has *the head of an ox;* or that any one who tells such a foolish story, has not *the head of an ass.* In this opinion I am not singular. Mr. Moore, in his excellent "Reply to the Antivaccinists," alluding to the ridiculous exhibitions in Saville Row, says, "This, however, had better be discontinued; for if the young pupils hear the Doctor striving to make them believe, in spite of their eyes, the transformation of the boy; when their imaginations are once roused, they may fancy they likewise see, solemnly discoursing, that head, which the mischievous mischievous fairy Puck so humourously clapt upon the shoulders of Bottom the weaver."

Dr. Rowley tells us he has exhibited cases of this sort at his lectures, before an audience of a hundred physicians, surgeons, apothecaries, and students. It is much to be wished, that the names of all those worthies, who countenanced such exhibitions, and impositions, were published. We should then probably see the names of gentlemen, who have emerged from their obscurity in Wapping and St. Giles's; the names of quacks, who have too long been suffered to murder his Majesty's liege subjects with impunity; and the name of a certain writer against vaccination, which shall not pollute my page. This man, I have been assured by the most unquestionable authority, was ready to commit perjury, on a certain occasion, in order to defraud a creditor, had he not been prevented.—I have been told, with some degree of emphasis, that the character of an author is of no consequence. Let any man judge for himself, what credit is due to an author, before the public tribunal, whose testimony would not be received in a court of justice. Let not the Antivaccinarian Society glory too much in this champion, who has committed so many *fundamental errors,* that no friends of Jenner, or of truth, will ever turn their backs upon him.

With respect to the formidable train of maladies ascribed by Dr. Rowley to the inoculated cow-pock, it is easy to prove that such an idea is totally destitute of reason. No such diseases are occasioned even by the casual cow-pox, which is much more severe; otherwise it would have been long since known in our dairies. It is also worthy of remark, that such complaints frequently occur in the families of the poor, where vaccination is only partially adopted; but seldom in those of the rich, where it is universally adopted. It is, indeed, the opinion of many practitioners of extensive observation, that the children of persons in the higher ranks were never so free from blemish as at this time.

The Editors of the Critical Review, in their account of Dr. Rowley's publication, condole with him, that he is "under the necessity of so repeatedly assuring the public of his long, very long, long experience in quarto and pamphlet, in Latin and English, in newspaper advertisements, and lastly on dead walls and deserted houses."—They observe, that his puffing hand-bills and advertisements, concerning his practice of half a century, are peculiarly culiarly unfortunate, when he writes concerning a disease, which eight years ago was unknown to the profession. They also justly observe, that Dr. Rowley's intention appears to be, not to elucidate the subject, "but, as the title page informs us, rather to supersede inquiry; to promote the reintroduction of the variolous pestilence; and to inform the world, that when this pestilence shall *most desirably* rage, Dr. Rowley is at hand with a 'certain, experienced, and successful method' of combating its virulence"

The Reviewers then remark, that in this contest, "the calm investigation of truth is impeded by every means which prejudice can suggest.—The most numerous, the most respectable, and the most respected members of the profession, in their zeal for the interests of humanity, relinquished with one accord a source of unceasing emolument; and adopted a practice which demanded little of their interference, and which spread no disease abroad to require their aid. Yet they have been stigmatised as acting from interested motives, by those men, who are averse from relinquishing the advantages of a practice, that is pregnant with misery to mankind."

They further add, "the character of this pamphlet, which is coarse and most inelegant in diction, may be comprehended from these general observations. Of the vulgar prejudices with which it abounds, in regard to beasts and 'beastly humours,' which from childhood to old age afford us most wholesome nutriment, we shall merely say, *that for him who is weak enough to entertain them, we have a sincere pity; and for him who urges them before the public, not believing them, a sovereign contempt.*

"Of the occasional pious cant and hypocritical sophistry, which is equally applicable to all inoculation, nay to all human attempts to alleviate human misery, we shall say nothing. Of the *facts* we must observe, that all those which have been before published, are again brought forward, though several of them are extremely doubtful, *and several absolutely disproved*; that others rest on the authority of *Doctor Squirrel, Mr. Roberts,* and other persons equally distinguished in the profession; and that several of them are mentioned without any reference or authority at all."

"The pamphlet will, indeed *signalise* its author; but the distinction will be such as we would not willingly see conferred on our decided enemy.—We would not willingly lingly say, "Oh that mine enemy would write *such a book!*"

The extraordinary claims of Dr. Rowley to superiority over his professional brethren, previously to the publication of his present pamphlet, have not escaped notice. I have now before me the Medical Review for April, 1802, from which it appears, that the Doctor's Treatise on the Watery-head of Children serves as a puffing advertisement, and savours so much of empiricism, that even truth itself would labour under a disadvantage, if brought forward in such a manner.

Dr. Rowley expresses a great dislike to innovations, and a great contempt for those who are less advanced in years than himself. In short, he thinks no man fit to practise physic till he is arrived at his dotage. It was not always so. Formerly the Doctor was young; then he endeavoured to disparage those who were older than himself. Now he is old; and endeavours to disparage those who are younger than himself.

In his letters to Dr. Hunter, he says, "It is an arduous task to remove prejudices, in men so self-sufficient and self-important as *some physicians are.—Men advanced in years, whose prejudices are habitual, who possess a plenitude of pride and practice,* have little time for reflection.—Endeavours of this nature are treated with supercilious contempt. *Any desires to improve the art, frequently excite envy in proportion to their merit; and are illiberally termed innovations.* They meet with every opposition which artifice can invent, or medical grimace furnish.

"I could produce several instances of the most exalted characters, who have been persecuted and detested by the faculty, for what has rendered their names immortal. I scarcely know any improvement in medicine, which has not met with strong opposition. It is a melancholy reflection, that a man should incur the displeasure of the members of his profession, for those very inventions, which merit the approbation and esteem of all mankind. Let us reflect for a moment on the fate of Vesalius and Harvey, and the introducers of chemical remedies, bark, antimony, and mercury; not to mention the unmerited opposition to your own discoveries in the lymphatic system."

"The common modes of practice are frequently so inadequate to the obstinacy of some diseases, that I am disposed to think, every rational attempt to improve our art is laudable.—We should always recollect *how much we*

*pretend to, and how little we comprehend, in the immense fields of medical science.*"

Here the Doctor was sensible, that there were persons in the world, who pretended to much more than they understood; and he was ready to include himself in the number. But it must be recollected, that he was not then "*a public lecturer on the theory and practice of medicine, excluding false systems, &c. &c.*"

In the Introduction to his Treatise on Sore Legs, he tells us, "prevailing prejudices are there censured, not perhaps with that degree of complacency, which readers of a certain class expect. Men habituated to a faithful and implicit observance of old doctrines, however false, bear with impatience any attempt to convince them of error. Accustomed in infancy to receive resemblances for realities, and opinions without demonstrations, and having passed, perhaps, a long life in error, *they are exasperated by an overthrow of their favourite systems.* The mysterious oracles of indolent tranquillity, and *specious deception,* must not be profaned with impunity by the sacrilegious hand of innovation:

Namque hoc tempore

Obsequium amicos, veritas odium parit.

"The more successful any improvement is," continues Dr. Rowley, "with so much the more fury it is opposed; as the history of medicine fully testifies, in the examples of mercury, bark, antimony, *inoculation,* &c. Every instance of an extraordinary cure reflects dishonour on the unsuccessful; and rarely fails to excite *in envious minds, private opposition, at the expense of honour, integrity, and truth.*"

On this occasion, the Doctor seems to have been quite a prophet. He has exactly foretold the part he was to act. We have not yet forgotten the report of the child of a wine merchant in Bond Street. The Doctor informed an anatomist, that this child died the preceding night of vaccination, when, in reality, he had died three weeks before of a watery head. This is one of the false reports noticed in my Treatise; and the refutation of it, as well as of many others in that work, is sufficient to prove the falsity of the assertion, so often repeated by the opponents of vaccination, that the friends of the practice had never instituted any inquiry, in order to ascertain whether their opinions were well founded or not.

Another

Another report propagated by Dr. Rowley, proved a considerable obstruction to the practice. This was concerning a child of Mr. Bignol, at the Wine Vaults, in the Broad Way, Westminster. It appears to be the thirty-first case alluded to by the Doctor in his pamphlet. He there says, "the child had a putrid malignant fever, and died of an universal gangrene." He takes care, however, not to tell the whole truth; otherwise, he would have told us, *that he never was inoculated for the cow-pock, but for the small-pox; that he had a confluent eruption, and died under his own care.*

About four years ago, I introduced Mr. Woolriche, Surgeon of the Naval Asylum, to Dr. Hooper, at the Mary-bone Infirmary; who offered to shew us the wards. Dr. Rowley, happening to arrive at the same time, accompanied us. Soon after we had entered one of the wards, Dr. Rowley abruptly introduced the subject of vaccination, and said, "If I were to tell Ring of a thousand cases of the small-pox after the cow-pox, he would not believe them." To this I replied, that "I should not believe them without better authority." One reason for this was, that I knew Dr. Rowley had not paid much attention to the subject, and did not understand it.

He then related the case beforementioned, as one instance of the cow-pock proving fatal. I asked where the parents resided. This he refused to answer. I then asked, who was the surgeon that vaccinated the child. He replied, "He should not tell me that. The surgeon had already been punished sufficiently; and had suffered much in his business and reputation." I then inquired, whether there had been any eruption. He replied, "there had been an eruption all over the body." I told him, this was a proof that the disorder was not the cow-pock. He said, he knew it to be the cow-pock, because it was black. I observed, that the arm might well be black, when in a state of mortification. He observed, that by looking into Dr. Jenner's publication, he saw the cow-pock was black. This, I informed him was a mistake. I then asked him, where the matter was obtained. He replied, "*Why, to be sure, the matter was obtained from Mr. Sutton for small-pox matter.*" This, I remarked, was fatal to his argument.

Having learned from Dr. Jenner, long ago, that Dr. Poignand, or Dr. Bradley, could inform me where the parents of this child lived, I called on Dr. Poignand soon after Dr. Rowley's pamphlet appeared, in order to investigate the case; and he accompanied me on the occasion.

 Mr.

Mr. and Mrs. Bignol told us, that their child, and a child of their partner, had been inoculated at the same time, by Mr. Braid, then residing in the Broad Way, Westminster, but now in Northumberland Street, Strand. He was desired to inoculate the children for the small-pox; and they had not entertained any doubt of his having done as he was desired, till Dr. Rowley was called in, who declared that they were inoculated with the cow-pock.

This was the more extraordinary, when it is stated, that both children had a confluent eruption. The partner's child had that species of small-pox called purples, yet recovered; contrary to Dr. Rowley's prediction, who declared it was impossible. Mr. Bignol's child, he said, might recover; but it died.

I have inquired the particulars of these cases of Mr. Braid; who declares that he inoculated both the children with the small-pox, and that they both had the confluent disease. He says, he had the matter, not from Mr. Sutton, but from Mr. Hanbury. Mrs. Bignol positively asserts, that her child's arm did not mortify, as stated by Dr. Rowley.

As a proof that the matter was not vaccine, Mr. Hanbury assures Mr. Braid, he took it from one of his own patients; and at that time he had not commenced vaccination.

This recalls to mind a false report of a similar kind, propagated by a medical man, concerning his own practice. When two of his patients, whom he had inoculated for the small-pox, had the natural disorder afterwards, he wished to transfer the blame to vaccination; declaring that he had inoculated them with cow-pock matter. Finding he could not justify his assertion, and knowing that vaccination was not practised at the time, he has since denied his words. Mr. and Mrs. Lowe, of Charles Street, Westminster, can verify the truth of this statement.

Such are the falsehoods and misrepresentations, by which the public have been deluded, from the commencement of vaccination to the present hour. Dr. Rowley enables us to account for this. In his Treatise before-mentioned he says, '*Improvers of arts are commonly treated with ingratitude;* and although mankind privately avail themselves of discoveries, *they commonly abuse the inventors. Calumny and detraction have been, and probably ever will be exercised, against every attempt to improve medicine; particularly in large cities, where men frequently become jealous competitors for extensive practice.*

Dr.

Dr. Rowley now looks with contempt on those who are younger than himself: formerly he looked with contempt on those who were older. He now reverences a hoary head; but, as far as we can judge from his writings, he never reverenced a hoary head, till there was a hoary head upon his own shoulders. He now tells the world to consider, whether impetuosity and youth are to be put in competition with hoary age. He asserts, that those who have had *the longest experience*, must be better qualified to judge on all important professional questions, than those who have had only ten or twenty years practice; hinting at the same time, what he is yet to prove, that there are old men whose intellects are vigorous and unbiassed; and that young men do not possess the same sort of mental powers as the old.

He did not always entertain this opinion. In his Essay on the Sore Throat, he says, "Experience alone, for ages, scarcely improved physic; nor does *the longest experience ever form a great physician. Old age often rivets the fallacies driven into the juvenile mind.*"

In the Introduction to his Memoir on "the Causes of the great Number of sudden Deaths amongst Adults and Children in Putrid Scarlet Fevers, &c. he says, '*It is a notorious fact, that many of the faculty grow white-haired, and bald-headed, in errors and prejudices; and when these die, there are others who are become grey-headed under the former professors.* These step into the cathedra, or professor's chair, and pursue the old beaten tracks; without ever reflecting that they are erroneous, or capable of improvement; and even if they perceive errors, they are too indolent to expose or attack them, but leave that task for those who follow in succession.

"*If, however, a man should not have sufficient penetration to discover, early in life, the defects of the medical art, and if he possess not a warm desire and spirit to remove them, he never will, in old age, attack, much less defeat, the hydra-headed monster of hereditary prejudice.*" "Hence," Dr. Rowley observes, "it is easy to see, whence, and by whom, improvements, however important, have been and are opposed." In the Introduction to his Essay on Sore Throat, he says, "The most important discoveries, which have elevated the medical art to its present respectability, have frequently been introduced *amidst the fury of party, and the hissings of envy.* A professional man, therefore, who has penetration to detect, and courage to expose error, or intrude new doctrine, however meritorious, has no

 more more right to expect confidence or candour, than his predecessors."

This, it must be confessed, is a very candid and honest declaration; and it will not be the fault of Dr. Rowley and his friends, if it be not verified in the present instance.

"Envy will merit, as its shade, pursue,
But like a shadow, proves the substance true:
For envy'd wit, like sol eclips'd, makes known
Th' opposing body's grossness, not its own.
When first that sun too pow'rful beams displays,
It draws up vapours, which obscure its rays;
But ev'n those clouds at last adorn its way,
Reflect new glories, and augment the day."

No one who reads the preceding extracts, will be at a loss to know, why Dr. Moseley, in his "Treatise on the Lues Bovilla, or Cow-pox," abuses the followers of Dr. Jenner for not suffering him "*to prosecute his discovery deliberately in the country;*" and "*to investigate it in a quiet philosophic manner,* through a succession of many experimental years. For, as Dr. Rowley justly observes, calumny and detraction ever have been, and probably ever will be, exercised against every attempt to improve medicine; *particularly in large cities, where men are jealous rivals, and competitors for extensive practice,*

It is no wonder, therefore, that Dr. Mosely inveighs against "cow-pox wizards" and medical jugglers," for snatching the raw material from the hand of Dr. Jenner, and disseminating it far and wide; to the great annoyance of certain members of the profession, and the great injury of trade.

When such attempts are made to undermine the practice of medicine, and explode the *old* profitable inoculation, the task of calumniating the *new* is, we are told, imposed on certain gentlemen "by *imperious* circumstances." It is much to be regretted, that among others, this task is imposed on Mr. Daniel Sutton; who has either written a letter to Dr. Moseley, or permitted Dr. Mosely to write one in his name, full of compliments to the Doctor, and invectives against the friends of vaccination. This letter, which was lately published in the Gentleman's Magazine, will remain as a lasting memorial of the sad necessity to which the enemies of vaccination are reduced, of trumpeting their own praise.

I am ready to acknowledge the merit of Mr. Sutton, in reviving the cool treatment of small-pox, as practised by the Arabians and Sydenham. Whether "imperious circumstances," or choice, caused him, late in life, to recommence commence inoculation, I do not presume to determine; but I have heard him say, it was very unfortunate for him, that soon after his retnrn to practice, Dr. Jenner published his account of the cow-pock. This expression appeared to me rather singular; nevertheless I was not willing to suppose, Mr. Sutton really regretted that this important discovery was made. Could any thing induce me to alter my opinion on that subject, it is the eagerness with which he has embraced an early opportunity of depreciating vaccination.

Mr. Sutton informed me, four years ago, that he had vaccinated forty-two persons, and that he never refused to inoculate for the cow-pock those who desired it. I am told, he has lately inoculated for the small-pox some relations of a violent opponent of the cow-pock, by whom he was recommended to the family. Whether gratitude for this favor, or any other motive, prompted him to take up his pen, or to suffer that gentleman to take it up for him, I cannot help offering him one word of advice, which is, not to provoke a controversy on this occasion. But should he despise this counsel, it may be thought necessary to draw a comparison between his conduct and that of Dr. Jenner; a comparison, which may not turn out much to his advantage. We shall then see a striking contrast between the two great improvers of inoculation; one of whom nobly and generously divulged the whole of his plan; the other divulged no part of *his* plan, which it was in his power to conceal.

---

I now beg leave to add a few miscellaneous observations. Mr. Moore has stated, that the younger brother of Jowles, whom Dr. Rowley calls the ox-faced boy, was inoculated with the small-pox. This is a mistake; he had the disorder in the natural way; and consequently the scrofula which ensued is the more likely to proceed from an hereditary taint, like that of his brother.

Dr. Henry Fraser has attempted to invalidate one of the cases of the small-pox a second time, which I published in this Journal. It is that of a son of Mr. King, in Printer Street, inoculated by Mr. Wachsel. Mr. and Mrs. King positively declare, that Mr. Wachsel was satisfied at the time, ordered the physic, and told them the child was safe. In opposition to such evidence, any doubt now expressed is of little avail. Mr. Ridout was also a witness to the marks remaining after the pustules; and affirms that Dr. Woodville acquiesced in the opinion of its being a recurrence of the disease.

Dr. Fraser seems to adopt the opinion of Dr. James Sims, that the cow-pox proceeds from the small-pox. Gentlemen who indulge themselves in this hypothesis, appear to be ignorant that it is not new. I long ago noticed it in my Treatise on the Cow-pox, as entertained by Dr. Turton, of Swansea. It requires, however, little reflection, to perceive the improbability of its being well founded. If the cow-pox originated from the small-pox, and were communicated to the cow by the hand of the milker, it would be as well known in Cheshire as in any other county; and be more or less prevalent in almost every part of the world.

That the disease called the genuine cow-pox proceeds from the horse, is as clearly proved as any other fact in medical science; a similar disease, endowed with a similar prophylactic virtue, having been repeatedly produced in the human subject, without the intervention of the cow. This, among other instances, has been done by Dr. Sacco, of Milan, who was before sceptical on the subject, in the presence of several medical men, assembled for that purpose; one of whom was a friend of Dr. De Carro. Matter of this kind has been transmitted to Dr. De Carro, and to Dr. Friese of Silesia; and has also succeeded in their hands. They now inoculate indiscriminately with equine or vaccine matter; and with equal affect. One of our Medical Journals, some months ago, announced, that another practitioner in Germany had also succeeded in an experiment with equine matter.

Dr. Sims also hazards an opinion, that the spurious cow-pox is the chicken-pox transfered from the human subject to the cow. This hypothesis rests on no better foundation than the other. The spurious cow-pock, in some instances, appears to be the effect of cold; and might be called a chilblain with as much propriety, as the affection which commonly goes by that name. The favus, when it happens on the face, and the vesications behind the ears, often arise from the same cause. I have seen this complaint on the nipples of cows every winter since the practice of vaccination in the metropolis commenced. One cow, at Camberwell, has had the disorder three years following, about the month of November. The last time it was more violent than on either of the former occasions. As to the duration of the disorder, that depends on different circumstances, such as the weather, the treatment, and the degree of injury occasioned by the hand of the milker.

Dr.

Dr. Jenner, in the first part of his "Inquiry," observes, that pustules of this sort frequently appear on the nipples of cows, but particularly in the spring, and when they are suckling their young.

Dr. Sims, after offering his conjecture, that the spurious cow-pox originates from the chicken-pox transfered to the brute animal, says, "this supposition easily accounts for a number of the failures recorded of vaccination, which have arisen from not accurately distinguishing one kind of pock from the other.

That the matter of the spurious cow-pock is incapable of preventing the small-pox, is allowed on all hands; it is therefore of little consequence from what source it originates. But by referring to the failures of vaccination which are on record, it will appear evident, that the matter in general was not procured immediately from the cow; and that they are not to be accounted for in this manner.

In the Medical and Chirurgical Review for January, Dr. Woodforde has published what is there called, a case of small-pox after casual cow-pock fifteen years before. Dr. Woodforde tells us, that when infected with the cow-pock, she had several eruptions, or pustules, in different parts of the body, which have left pits or cicatrices. This corresponds much more with the idea of the chicken-pox than that of the cow-pox. Mr. Giffard, of Gillingham, long ago, informed us, that *in some places, the chicken-pox, or swine-pox, is called by the lower orders of people the cow-pox.* He says, *there are two kinds of cow-pox; one is attended with eruptions on the skin in general, and sometimes produces pits; the other is a disease confined to the hands.* This induced me to make the following observations in my Treatise on the Cow-pox, p. 45. "From the preceding accounts it must appear evident, that several cases of cow-pox, inserted in the different Journals and Magazines, were of the spurious kind. In that published by Dr. Hooper, in the London Medical Review for July, 1799, we are told, the patients had eruptions on different parts of the body; whereas those of the genuine disease are local."

It must be acknowledged, that a cow-pock may be excited in any other part of the body, besides the hands, in consequence of the insertion of matter; but I do not recollect any authentic instance of the disorder, in which it bore the character of that described by Dr. Woodforde.

It

It is reasonable to suppose, that when the vesicles in the true cow-pox are ruptured by friction, the matter which is secreted undergoes a gradual change, and that it is capable of producing a disease, when it is no longer capable of preventing the small-pox. This is agreeable to the opinion of Dr. Jenner, who observes, that a person who milks a cow one day may receive the genuine infection and be secured, and that another person, who milks the cow the next day, may still be liable to this dreadful distemper.

Dr. Jenner very properly says, "what confusion should we have, were there no other mode of inoculating the small-pox, than handling the diseased skin of a person labouring under that distemper in its advanced stage?" It is well known, that when variolous matter has been taken at this period, and inserted, the most violent inflammation has ensued, attended with abscesses and eruptions; yet the patient has still proved susceptible of the small-pox.

This woman, it seems, was so confident of her security from the contagion, that she had never hesitated visiting any person labouring under it; and, in fact, she had repeatedly done so with impunity. This, together with other similar instances, appears to countenance the idea of a diminished susceptibility of variolous infection.

Dr. Woodforde remarks, that the foregoing case, together with some other of the same kind, must certainly arrest the attention of every impartial person, and excite some suspicion of the permanent preventive power of vaccination. A similar doubt of the permanent preventive power of the small-pox was long ago expressed by Dr. Adams, in his Treatise on Morbid Poisons; and this doubt also is countenanced by a number of cases equally strong.

Dr. Woodforde observes, that in forming principles of any kind, inattention or indifference on one hand, or sanguine bigotry and enthusiasm on the other, must be equally adverse to success. On the present subject, he fears we have most to apprehend from the latter; and Dr. Rowley is of the same opinion.

In the same Number of the Medical and Chirurgical Review, is an Extract from the Minutes of the Committee of the Vaccine Pock Institution, in which is the following remark. "In this case we have a proof of what has been already asserted at this Institution, that variolous matter can exert its agency upon the constitution, at a period of the vaccine process in which it has been supposed that the variolous insusceptibility has been produced. But it seems reasonable to conclude, that the anti-variolating process is of several days duration." In one case there adduced, the eruption of the small-pox appeared on the thirteenth, and in another on the fifteenth day. In my Treatise on the Cow-pox, I long ago gave an account of its appearing at a much later period.

When speaking of Dr. Henry Fraser's publication, I forgot to mention one remark of his, which is rather singular in any man who attempts to write on the subject of vaccination. After giving his opinion in favor of a second inoculation during the progress of the first, he says, it appears to him that Mr. Pearson was the first who ever made such an experiment; and that he discontinued it in compliance with the opinion of some eminent vaccinators, who unreasonably suggested, that it might prove inimical to the establishment of the practice; and because, like a true philanthropist, he was unwilling to offer any impediment to the progress of so beneficial a discovery.

I cannot but be a little surprised, that Dr. Fraser should so readily suppose Mr. Pearson to be the first who made this experiment, without giving some reason for that supposition; and without noticing any of the authors or other practitioners who have done the same. It is well known to those who are conversant in the history of Vaccination, that the most eminent inoculators have occasionally practised it from an early period; and that it was even practised in the old method of inoculation. Mr. Bryce particularly recommended it four years ago; and Mr. Hugo, who had long practised it, has since recommended it also.

Mr. Pearson spoke to me on the subject, about fifteen months since. I know not who may be the eminent vaccinators alluded to by Dr. Fraser; but I am one of the humble vaccinators who suggested what Dr. Fraser deems so unreasonable. In justice, however, to myself, and others who suggested the same apprehension, it must be remarked, that at the time of this transaction, only the Fullwood's Rents cases had occurred to stagger our faith. I have since communicated my sentiments on the subject in this Journal.

I am, &c.

